# Retrograde Type A Aortic Dissection With a Double Aortic Valve Sign Presenting As Acute Coronary Syndrome: A Case Report and Literature Review

**DOI:** 10.7759/cureus.101856

**Published:** 2026-01-19

**Authors:** Karim M Elzoghby, Raffi Almutawa, Rania S Ahmed, Salah N El-Tallawy, Joseph V Pergolizzi, Abdullah T Alsubaie, Giustino Varrassi

**Affiliations:** 1 Cardiology, Saudi German Hospital Riyadh, Riyadh, SAU; 2 Cardiology, Alfaisal University, Riyadh, SAU; 3 College of Medicine, Alfaisal University, Riyadh, SAU; 4 Anesthesia and Pain Management, Faculty of Medicine, National Cancer Institute (NCI) Cairo University, Cairo, EGY; 5 College of Medicine, King Saud University, King Abdulaziz University Hospital, Riyadh, SAU; 6 Pain Management, NEMA Research Inc., Naples, USA; 7 Anesthesia, King Abdulaziz University Hospital, Jeddah, SAU; 8 Pain Medicine, Paolo Procacci Foundation, Rome, ITA

**Keywords:** acute aortic dissection, acute chest pain, atypical presentation of aortic dissection, double aortic valve sign, misdiagnosed acute coronary syndrome, misdiagnosed aortic dissection, retrograde type a acute aortic dissection, retrograde type a dissection

## Abstract

Acute type A aortic dissection (ATAAD) is a time-sensitive and life-threatening emergency that usually presents as sudden, severe pain radiating to the back. Atypical presentations such as acute coronary syndromes make it challenging to diagnose, hence delaying the management.

We report a case of a 57-year-old male with a history of hypertension, diabetes, dyslipidemia, and chronic smoking who presented to the emergency department with central chest heaviness that radiated to the left hand associated with diaphoresis. Normal cardiac enzymes and an electrocardiogram (ECG) led to the initial diagnosis of unstable angina. As a result, he was given dual antiplatelets, which provided temporary relief of his symptoms. However, before discharge, a bedside transthoracic echocardiography found a retrograde ATAAD with the double aortic valve sign, a rare echocardiographic finding, along with severe aortic regurgitation. Further imaging with CT angiography confirmed the diagnosis despite the patient not complaining of any pain. Due to the involvement of the aortic root, valve, and right coronary artery, he underwent the Bentall procedure. Postoperative management emphasized initiation of anticoagulation, controlling the blood pressure, and management of cardiovascular risk factors to prevent aortic dissection and aneurysm. The patient recovered from the postoperative course without complications and remained stable upon follow-up.

ATAAD should remain in the differential diagnosis for acute chest pain even in the absence of a typical presentation. Bedside echocardiography is a useful initial diagnostic tool, and early multidisciplinary management supports successful patient outcomes.

## Introduction

Acute aortic dissection (AAD), a rare yet life-threatening cardiovascular emergency, involves the separation of the intima and media layers of the aorta, creating a false lumen that can extend proximally, distally, or retrograde, compromising blood supply to vital organs [1]. Aortic dissection (AD) is classified according to the Stanford System into Type A, in which the aorta must be involved and requires immediate surgical intervention, and Type B, in which the descending aorta alone is involved, requiring medical management with close follow-up and, in some cases, surgery [1,2]. Some risk factors for AD include male gender, patients above 60 years old, hypertension, atherosclerosis, aortic aneurysm, bicuspid aortic valve, coarctation of the aorta, cocaine use, third-trimester pregnancy, vasculitides such as giant cell arteritis, and congenital conditions such as Turner’s, Marfan, type IV Ehlers-Danlos, and Loeys-Dietz syndromes [3].

Despite the presence of a sudden onset of severe chest pain radiating to the back, AD can present atypically with syncope, stroke, shock, heart failure, cardiac tamponade, lower limb ischemia, signs and symptoms of end-organ failure due to compromise in the blood flow, or even be painless [1]. Due to the overlapping signs and symptoms of AAD with other cardiovascular conditions, around 30% of the patients diagnosed with AAD were initially misdiagnosed with another condition [4]. In fact, around 50% of the misdiagnosed cases of AD were acute coronary syndrome (ACS), highlighting the importance of prompt, correct diagnosis and management since the management of ACS is dual antiplatelet therapy, while it is prompt surgical intervention for acute type A aortic dissection (ATAAD) [1,5]. As a result, giving dual antiplatelets to a misdiagnosed ATAAD patient could lead to catastrophic outcomes, such as significantly increasing the risk of bleeding that might lead to shock if caused by delaying the surgical management of ATAAD [6]. In fact, the mortality rate of ATAAD rises by 1-2% per hour within the first 24-48 hours and reaches up to 50% within 48 hours if left untreated [1].

CT angiography is the primary diagnostic imaging modality for AAD, with a sensitivity and specificity reaching up to 98-100% [7]. However, echocardiography, although operator dependent, plays a vital role in the diagnosis of ADD, especially in areas where advanced imaging modalities such as CT angiography are unavailable. The presence of the "double aortic valve sign," first described by Loumiotis et al., where the intimal flap gives rise to the appearance of a second aortic valve leaflet, is a significant finding on echocardiography in retrograde ATAAD [8]. This finding is very rare to the extent that we were able to find only four reported cases on PubMed of patients with this sign [8-11].

We report a rare case of retrograde type A aortic dissection (TAAD) in a middle-aged man who initially presented with signs and symptoms indicative of unstable angina, but was subsequently diagnosed with retrograde TAAD characterized by a double aortic valve sign.

## Case presentation

Ethical standards

Written informed consent was obtained from the patient for publication of this case report. The CARE guideline checklists (for CAse REports) were followed in the preparation of this manuscript [12].

Initial presentation

A 57-year-old male with diabetes, hypertension, dyslipidemia, and a history of heavy smoking presented to the emergency room of a rural hospital, complaining of sudden onset, continuous central chest pain rated 8/10, by verbal rating scale described as heaviness/ tightness in the chest. In addition, the pain radiated to the left arm and was associated with diaphoresis. The pain started 20 minutes before presentation with no exacerbating or relieving factors. In addition, the patient denied any symptoms of palpitations, dizziness, syncope, or radiation of the pain to the back or jaw.

The patient was diagnosed with hypertension 10 years ago and has been taking bisoprolol 2.5 mg daily, spironolactone 25 mg daily, and valsartan 80 mg twice daily. He has been diabetic for one year and is on metformin 1000 mg twice daily. For the dyslipidemia, he is on atorvastatin 40 mg once per day. The patient's diabetes, hypertension, and dyslipidemia are controlled, and he is compliant with all the medications. The patient denied any history of any heart disease or any other medical condition, despite the ones mentioned. He has a family history of diabetes and hypertension; however, no history of any cardiovascular or genetic diseases, sudden cardiac death, or aortic aneurysms or dissection.

The patient consumes a regular diet and engages in mild physical activity. However, he has been a heavy smoker, smoking two packs per day for the past 37 years. The review of systems was insignificant.

Initial evaluation

The patient’s vital signs showed hypertension only, as shown in Table 1.

**Table 1:** Vital signs of the patient

Vital signs	Patient’s values
Heart rate	76 beats per minute
Blood pressure	140/90 mmHg
Temperature	37.2°C
Respiratory rate	18 breaths per minute
Oxygen saturation	98% on room air

Physical examinations were all within normal limits, as seen in Table 2.

**Table 2:** Physical examination findings of the patient

System	Findings
General appearance	The patient was awake, oriented to person, place, and time. However, he showed signs of pain but no signs of respiratory distress. Capillary refill was less than two seconds. All pulses were palpable, regular in rhythm, of normal volume, and symmetrical bilaterally. Pupils are equal, symmetrical, and reactive to light. Moist mucus membranes.
Cardiovascular	S1+S2 no added sounds or murmurs across all four valves.
Pulmonary	Equal and bilateral breath sounds.
Abdomen	Abdomen soft and lax with active bowel sounds. No pulsatile abdominal mass, usually felt in the epigastric area or at or above the umbilicus.
Neurological	Cranial nerves (CN) II-VI appears to be normal. No motor or sensory deficits.
Lower limbs	No lower limb edema or calf tenderness.

Diagnostic ECG, labs, and imaging

**Figure 1:**
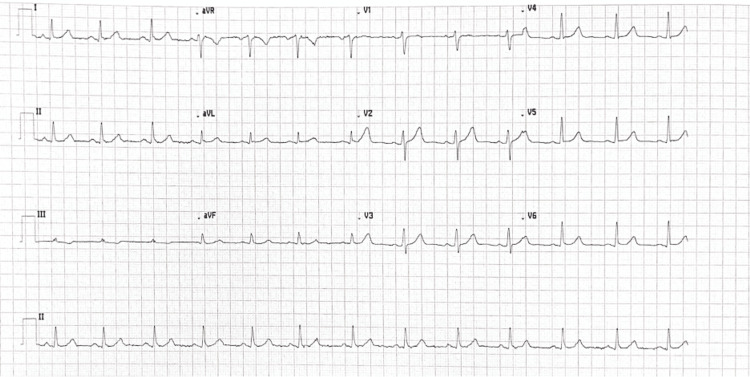
Electrocardiogram of the patient showing normal sinus rhythm

The electrocardiogram (ECG) showed a normal sinus rhythm, as seen in Figure 1.

Labs were all within normal limits, as illustrated in Table 3.

**Table 3:** Laboratory studies of the patient CBC: complete blood count; Hb: hemoglobin; PT: prothrombin time; PTT: partial thromboplastin time; INR: international normalized ratio; CK-MB: creatine kinase-MB; LDL: low-density lipoprotein; HDL: high-density lipoprotein; HbA1c: glycated hemoglobin

Name of the Test	Patient’s Value	Reference Value
CBC		
Hb	14.6 g/dL	12-16 g/dL (F), 13-17 g/dL (M)
WBC	5×10^9^/L	4.0-11.0×10^9^/L
Platelets	350×10^9^/L	150-400×10^9^/L
Coagulation Profile		
PT	13 sec	11-15 sec
PTT	28 sec	25-35 sec
INR	0.9	0.8-1.2
Cardiac Enzyme		
Troponin I	8 ng/L	<14 ng/L
CK-MB	1 ng/mL	<5 ng/mL
Lipid Profile		
Total Cholesterol	1.73 mmol/L	<5.2 mmol/L
LDL	0.78 mmol/L	<2.6 mmol/L
HDL	0.86 mmol/L	>1.0 mmol/L
Triglycerides	2.02 mmol/L	<1.7 mmol/L
D-Dimer	0.3 µg/mL	<0.5 µg/mL
HbA1c	6.6%	<5.7%

After reviewing the labs and ECG, the patient was admitted to the ICU as a case of unstable angina and given 300 mg aspirin as well as 300 mg clopidogrel, which provided pain relief within 45 minutes. His blood pressure and heart rate went down to 112/78 mmHg and 78 beats per minute, respectively, after the pain subsided. He was kept for observation overnight.

**Figure 2:**
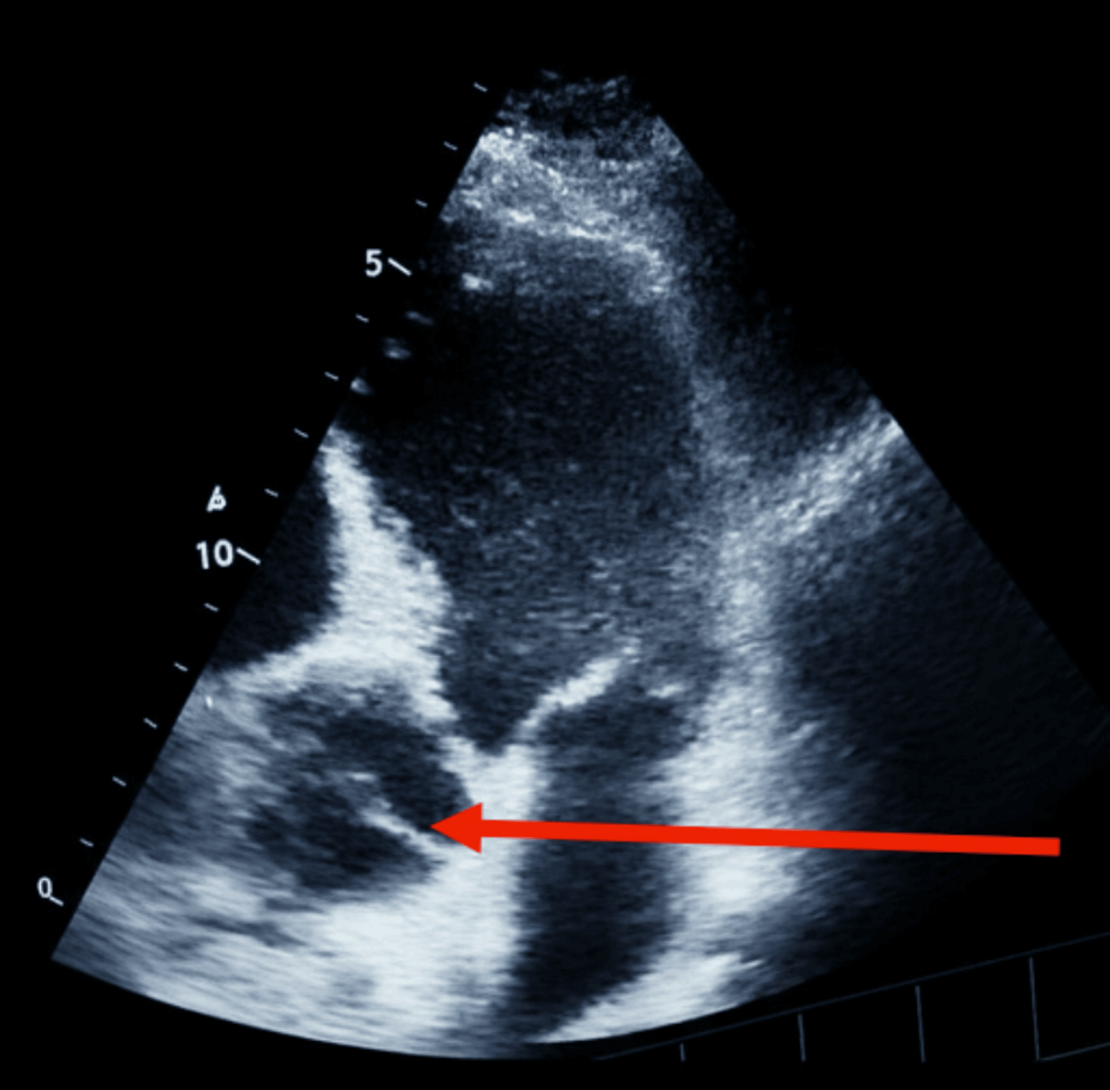
Two-dimensional apical four-chamber transthoracic echocardiogram showing a dilated ascending aorta and a dissection flap (arrow)

**Figure 3:**
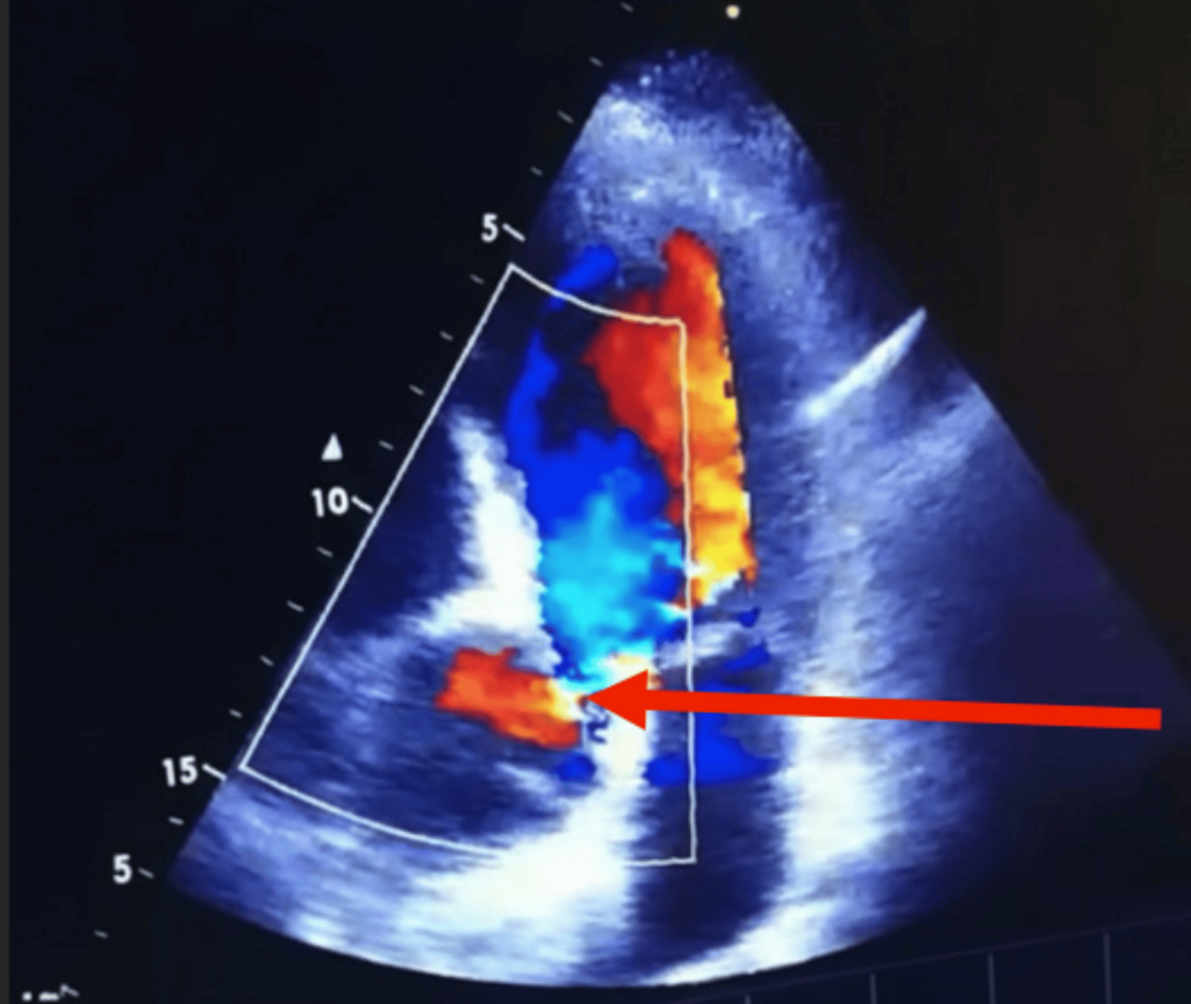
Two-dimensional apical four-chamber view on transthoracic echocardiography with Doppler demonstrating aortic regurgitation (arrow) The red arrow indicates the aortic valve, and the blue mosaic jet represents retrograde flow from the aorta into the left ventricle during systole, consistent with aortic regurgitation.

**Figure 4:**
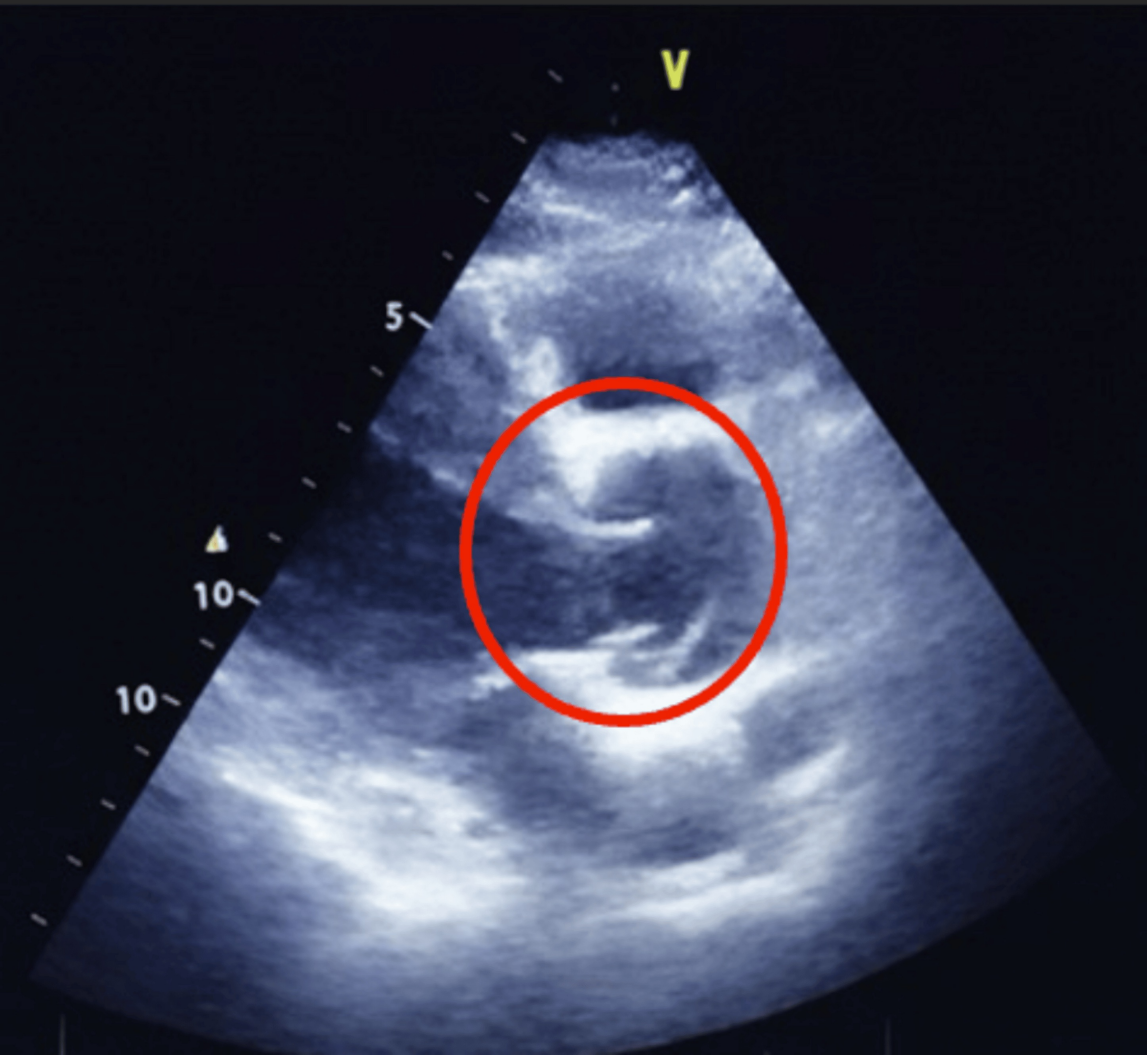
Two-dimensional parasternal long-axis view on transthoracic echocardiogram showing the double aortic valve sign (red circle)

Before discharge, a bedside transthoracic echocardiogram (TTE) revealed retrograde AD with double aortic valve sign involving the ascending aorta and severe aortic regurgitation despite not complaining of any pain. Figures 2-4 demonstrate these findings.

**Figure 5:**
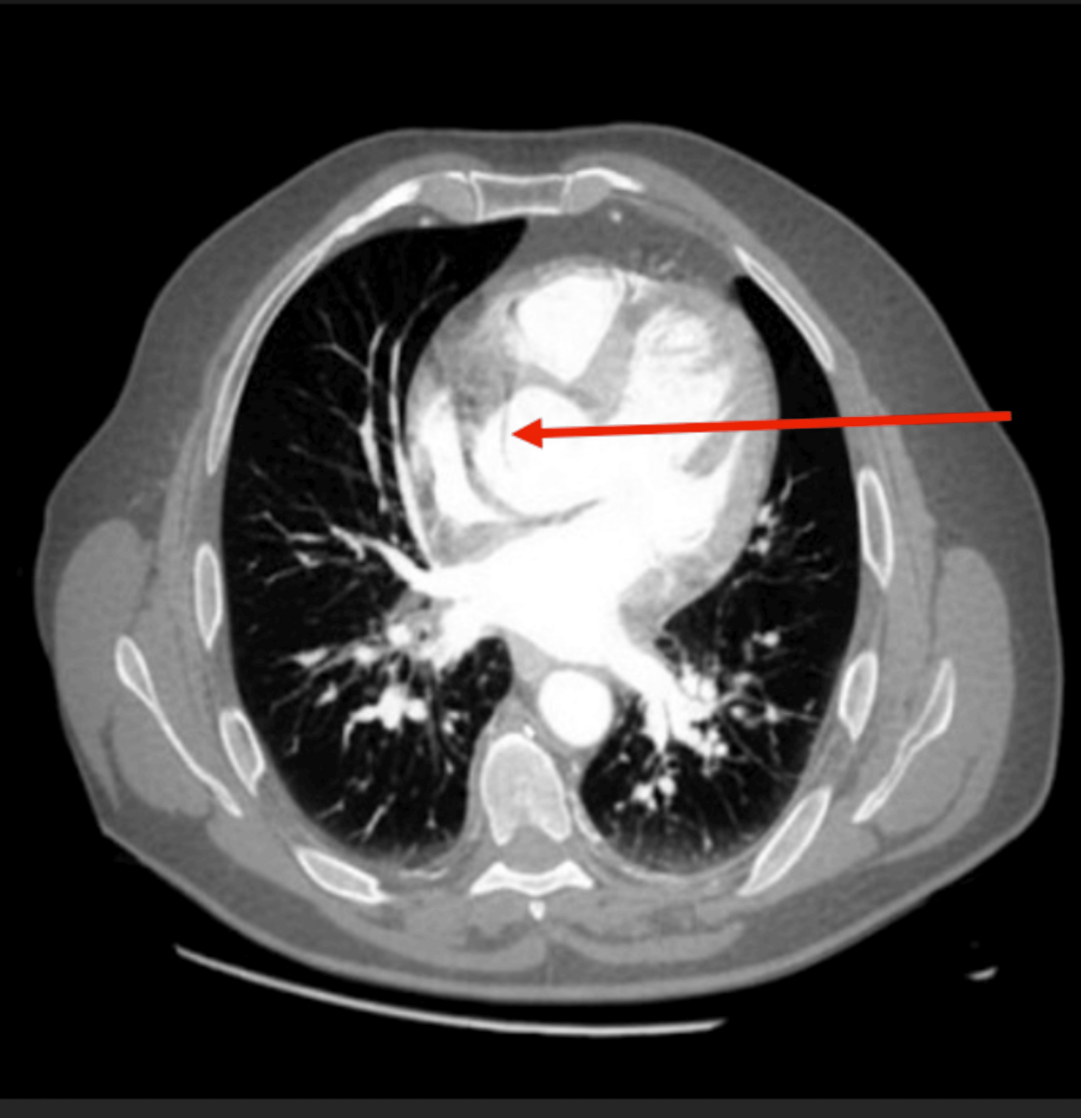
Axial contrast-enhanced CT aortography showing a dilated ascending aorta measuring 4.3 cm in diameter with an aortic dissection flap; the arrow indicates the site of dissection

After the TTE findings, the patient was rushed to CT aortography, which revealed a dilated supravalvular ascending aorta measuring 4.3 cm with an intimal flap, as seen in Figure 5. The aortic arch, thoracic, and abdominal aorta had normal caliber with mild atherosclerotic plaque.

The patient was vitally stable with a blood pressure of 112/78 mmHg and reported no pain. As a result, he did not receive any beta-blockers or analgesia while preparing the patient for transfer to a tertiary center for definitive surgical management.

Operative management

Intraoperatively, the right coronary artery (RCA) ostium was found to be compromised due to the extension of the dissecting flap. The decision to undergo the Bentall procedure was taken. The procedure involved replacing the diseased aortic root, ascending aorta, and aortic valve with a 27 mm mechanical valve conduit. The left coronary artery was directly reimplanted, while a saphenous vein graft was used to bypass the RCA because the RCA was dissected during the initial attempt to repair it and attach it to the conduit. There were no complications during or after the procedure.

Postoperative management

Postoperatively, the patient was admitted to the cardiac ICU for five days and then to the ward for three days for close monitoring and observation.

After that, he was discharged from the hospital on warfarin, 2 mg once daily, spironolactone, 25 mg once daily, atorvastatin 40 mg once daily, bisoprolol 2.5 mg once daily, esomeprazole 20 mg once daily, aspirin 100 mg once daily, paracetamol 1000 mg three times daily, metformin 1000 mg twice daily, and valsartan 80 mg, taken twice daily. In addition, he was asked to follow up with cardiology after three days, with labs for checking his international normalized ratio (INR), and cardiac surgery after 14 days. The patient showed up to the follow-up appointments with no new complaints and had an uneventful recovery.

## Discussion

ATAAD classically presents the sudden onset of severe chest pain that radiates to the back. However, atypical presentations occur in 6-17% of cases, leading to misdiagnosis and inappropriate management [13,14]. In our case, a 57-year-old male patient with multiple cardiovascular risk factors presented with central chest heaviness with radiation to the left arm associated with diaphoresis, initially diagnosed as ACS. Due to the initial improper diagnosis, the patient was managed with dual antiplatelet therapy that alleviated his symptoms, but led to a delay in diagnosing and receiving the appropriate management for ATAAD. This plays a crucial role in considering ATAAD as a differential for emergencies, despite the absence of classic symptoms.

Additionally, it is important to maintain a high suspicion for ATAAD in patients presenting with chest pain and initially managed as ACS, particularly in high-risk patients with a suggestive clinical course and image findings.

A key diagnostic tool that guided us to the diagnosis of ATAAD was TTE. While CT angiography remains the gold standard for diagnosing ATAAD, TTE offers a rapid, noninvasive bedside evaluation [15,16]. Echocardiographic findings such as the double aortic valve sign, aortic root dilation, severe aortic regurgitation, and pericardial effusion served as important indicators of ATAAD and led to appropriate management. Our case highlights the importance of TTE as a rapid, cheap, life-saving diagnostic tool in the emergency setting, particularly in hospitals where access to CT angiography is limited.

Once the diagnosis was confirmed by CT angiography, the patient was promptly prepped and transferred to a tertiary center. Based on the ultrasound finding of severe aortic regurgitation and the intraoperative finding of involvement of the RCA, the patient underwent the Bentall procedure. This procedure involved the replacement of the aortic root, valve, and ascending aorta and reimplantation of the coronary arteries. Although aggressive, the Bentall procedure remains the gold standard approach for ATAAD with valvular and/or coronary involvement [17]. Postoperative management included warfarin for the mechanical valve and highlighted the importance of strict control of blood pressure and lipid profile to avoid late complications such as recurrent dilation or dissection after successful repair. This highlights the importance of creating an individualized multidisciplinary approach and maintaining close follow-up to ensure an uneventful, successful recovery.

## Conclusions

This is a case of a 57-year old male with a background of hypertension, diabetes, dyslipidemia, and chronic smoking, presenting to the emergency department with symptoms of central chest heaviness radiating to the left hand and with associated diaphoresis. Further workup showed normal cardiac enzymes as well as a normal ECG. As a result, the patient was labeled as a case of unstable angina and provided dual antiplatelet therapy, which provided relief; however, bedside TTE before discharge revealed the double aortic valve sign, consistent with retrograde ATAAD, which was confirmed with CT angiography, and the patient was managed appropriately.

This case emphasizes several important points. First, ATAAD should always be considered as a top differential for any patient presenting with chest pain, even in the absence of the typical presentation, to avoid misdiagnosing and delaying management. Second, TTE serves as an important preliminary diagnostic tool in resource-limited settings, particularly in settings where access to CT angiography may be limited. Third, early recognition and timely involvement of a multidisciplinary team are cornerstones for having a successful recovery for patients with ATAAD.
